# Sex dimorphism in vascular trajectories after living-donor kidney transplantation: a prospective exploratory cohort study

**DOI:** 10.1093/ckj/sfag382

**Published:** 2026-10-27

**Authors:** Camillo Tancredi Strizzi, Samsul Arefin, Leah Hernandez, Awahan Raheman, Francesco Pesce, Giuseppe Grandaliano, Peter Stenvinkel, Karolina Kublickiene

**Affiliations:** Division of Renal Medicine, Clinical Science, Intervention and Technology (CLINTEC), Karolinska Institutet, Stockholm, Sweden; Department of Translational Medicine and Surgery, Università Cattolica del Sacro Cuore, Rome, Italy; Nephrology, Dialysis and Transplantation Unit, Fondazione Policlinico Universitario A. Gemelli IRCCS, Rome, Italy; Division of Renal Medicine, Clinical Science, Intervention and Technology (CLINTEC), Karolinska Institutet, Stockholm, Sweden; Division of Renal Medicine, Clinical Science, Intervention and Technology (CLINTEC), Karolinska Institutet, Stockholm, Sweden; Division of Renal Medicine, Clinical Science, Intervention and Technology (CLINTEC), Karolinska Institutet, Stockholm, Sweden; Department of Translational Medicine and Surgery, Università Cattolica del Sacro Cuore, Rome, Italy; Division of Renal Medicine, Ospedale Isola Tiberina-Gemelli Isola, Rome, Italy; Department of Translational Medicine and Surgery, Università Cattolica del Sacro Cuore, Rome, Italy; Nephrology, Dialysis and Transplantation Unit, Fondazione Policlinico Universitario A. Gemelli IRCCS, Rome, Italy; Division of Renal Medicine, Clinical Science, Intervention and Technology (CLINTEC), Karolinska Institutet, Stockholm, Sweden; Division of Renal Medicine, Clinical Science, Intervention and Technology (CLINTEC), Karolinska Institutet, Stockholm, Sweden

**Keywords:** arterial stiffness, endothelial dysfunction, kidney transplantation, sex differences, vascular aging

## Abstract

**Background:**

Cardiovascular risk remains high after kidney transplantation, reflecting pre-transplant early vascular aging. Arterial stiffness generally improves, but the trajectory of microvascular endothelial function, and its dependence on recipient’s biological sex and donor matching, is unclear.

**Methods:**

In 71 living-donor kidney transplant recipients, microvascular endothelial function (reactive hyperemia index, lnRHI) and arterial stiffness [augmentation index at 75 bpm (AI@75)] were measured before transplantation and at a median of 24.6 months. Forty-six of their donors were assessed identically before donation, providing a healthy reference of the same sex. Resistance arteries from 37 recipients and 46 living donors underwent wire myography.

**Results:**

AI@75 improved overall (−5.7%; *P* = .003), whereas microvascular function declined in men but not women (male ΔlnRHI −0.149, *P* = .002; sex × time interaction *β* = −0.19, *P* = .026), stable in direction across 11 specifications including tacrolimus exposure and graft function. The greater AI@75 improvement with sex-matched transplantation (*P* = .020) did not survive adjustment for baseline stiffness (*P* = .32). *Ex vivo*, endothelial relaxation showed no detectable sex difference in recipients (*P* = .83) or donors (*P* = .94), whereas passive stiffness was higher in female recipients (*P* = .048).

**Conclusions:**

After living-donor kidney transplantation, arterial stiffness improved in both sexes, while microvascular endothelial function declined in men and not in women. Referenced to healthy donors of the same sex, arterial stiffness in men remained abnormal at follow-up. Sex-matched transplantation conferred no independent vascular benefit. These findings are exploratory and require confirmation in larger cohorts.

## Graphical Abstract

**Graphical Abstract:**
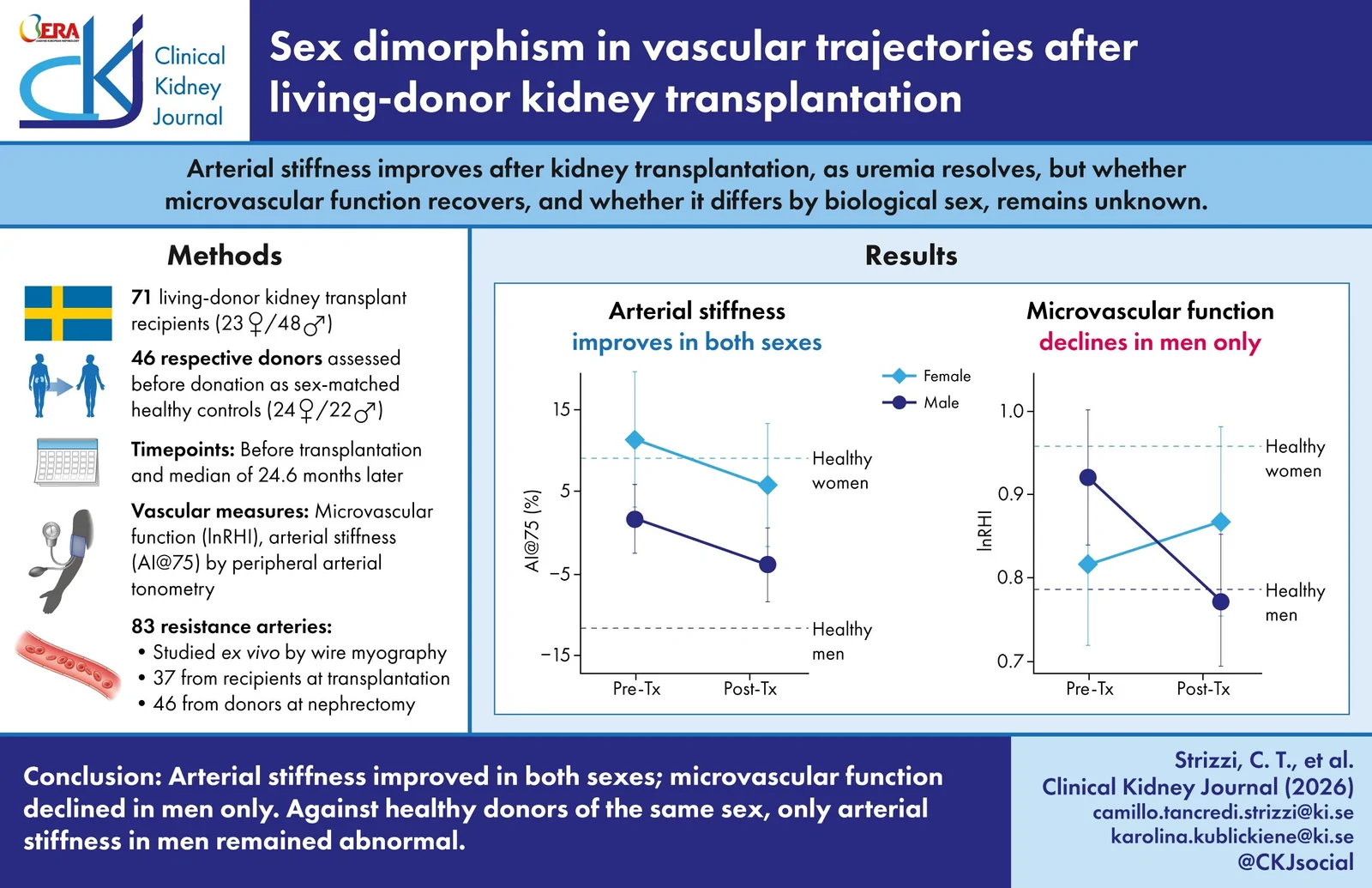

KEY LEARNING POINTS **What was known**:

- Arterial stiffness generally regresses after kidney transplantation, whereas the trajectory of microvascular endothelial function is heterogeneous and its dependence on recipient sex had not been examined.
- Biological sex shapes vascular physiology and transplant outcomes, and calcineurin-inhibitor vascular toxicity may be sex-dependent.
- Whether donor–recipient sex matching influences recipient vascular remodeling was untested.

**This study adds**:

- Over a median of 24.6 months, the two vascular compartments followed different courses: arterial stiffness improved in both sexes, while microvascular endothelial function declined in men and not in women.
- Assessed against their own living donors, male recipients remained stiffer than healthy men at follow-up.
- Tacrolimus exposure did not differ between the sexes and did not predict change; donor–recipient sex matching conferred no vascular benefit once baseline stiffness was accounted for.

**Potential impact**:

- Against healthy donors of the same sex, arterial stiffness in male recipients remained abnormal at follow-up, whereas their microvascular function did not.
- Reference values for both measures are sex-specific, and comparisons that ignore this may misclassify.
- These findings are exploratory and do not justify sex-differentiated post-transplant surveillance.

## INTRODUCTION

Cardiovascular disease remains the leading cause of mortality in kidney transplant recipients, accounting for 30%–50% of deaths with a functioning graft [1–3]. Although transplantation reduces cardiovascular event rates compared to dialysis [4], residual risk far exceeds the general population’s [5], reflecting incompletely reversible pre-transplant vascular damage and new insults from immunosuppression [6, 7].

This residual burden is increasingly framed as early vascular aging: through persistent inflammation, oxidative stress, and uremic toxin accumulation, chronic kidney disease drives a senescent vascular phenotype that accelerates micro- and macrovascular dysfunction [8, 9]. It comprises two interrelated pathologies, arteriosclerotic stiffening of large elastic arteries and microvascular endothelial dysfunction from impaired nitric oxide (NO)-mediated vasodilation [10, 11], captured by distinct metrics: pulse-wave velocity measures conduit stiffness directly, whereas the augmentation index reflects the timing and magnitude of the returning pressure wave, which depends on stiffness together with body size and heart rate. The reactive hyperemia index (RHI) from peripheral arterial tonometry captures acute, in part NO-dependent microvascular reactivity [12–14]. Because this phenotype is established before transplantation, a central question is whether it resolves uniformly across compartments and irrespective of sex, given its sex-dependent experimental features [8].

Meta-analysis confirms that arterial stiffness generally improves after transplantation [15]. The trajectory of microvascular function, however, is heterogeneous and incompletely characterized [16]. Our group previously found, in a small cohort, that vessel stiffness improved while RHI worsened at 2 years [17], suggesting that uremia correction and immunosuppressive exposure exert divergent effects on different vascular beds.

Biological sex is a fundamental determinant of vascular structure, function, and disease susceptibility [18, 19]. These differences are present in health, and community-based studies define abnormal digital vascular function using sex-specific thresholds derived from risk-factor-free reference samples [14]; the cut-off in common clinical use (RHI < 1.67) is not sex-specific. In transplantation, registry analyses show complex, age-dependent biological sex differences in graft and patient survival, with evidence that recipient’s estradiol, not chromosomal sex per se, modulates alloimmune responses [20–22]. Donor–recipient sex matching adds a further dimension, though reported associations with graft outcomes are inconsistent [23–26] and its effect on recipient vascular remodeling has never been examined.

Several determinants of the post-transplant vascular course could act in a sex-dependent way, including the vasculo-toxicity of calcineurin inhibitors [27, 28], sex differences in tacrolimus pharmacokinetics [29, 30], and hormonal and sex-chromosome-linked differences in endothelial signaling [31–33]. Whether the vascular trajectory itself differs by sex has not been established.

Existing evidence also relies on *in vivo* surrogates: RHI reflects the microvasculature within the systemic, hormonal, and pharmacological milieu and cannot isolate the vessel wall. *Ex vivo* wire myography of isolated resistance arteries addresses this directly, quantifying endothelium-dependent and -independent relaxation and partitioning the former into NO and endothelium-dependent hyperpolarization (EDH) components; such properties are themselves prognostically consequential [34, 35].

A further limitation of existing evidence is interpretive. Whether a given post-transplant value represents recovery, residual injury, or normality cannot be judged without an appropriate comparator, and published reference values are typically derived from unrelated populations and applied without regard to sex. Living kidney donors offer a near-ideal reference: they are rigorously screened healthy individuals, frequently genetically related to the recipient, and can be assessed with the same equipment and protocol at the same visit. Their vascular phenotype has not previously been used to calibrate post-transplant trajectories.

To our knowledge, sex-stratified trajectories of endothelial function and arterial stiffness after kidney transplantation have not been examined, nor has it been tested whether donor–recipient sex matching is associated with vascular change independent of baseline status. In this prospective single-center living-donor cohort with paired tonometry before transplantation and at a median of 24.6 months thereafter, we addressed three questions: whether the two vascular compartments follow different trajectories by recipient sex; whether donor–recipient sex matching influences vascular change; and how recipient values before and after transplantation compare with healthy donors of the same sex. We additionally used wire myography, on tissue obtained at transplantation surgery, to examine whether baseline resistance-artery function differed between men and women. Given the cohort size and the number of subgroups examined, these analyses are exploratory and intended to be hypothesis-generating.

## MATERIALS AND METHODS

### Study design and population

This prospective, single-center cohort study was conducted at Karolinska University Hospital, Stockholm, Sweden. We enrolled consecutive adults (≥18 years) with end-stage kidney disease scheduled for living-donor kidney transplantation between January 2017 and March 2024. Seventy-one recipients completed paired peripheral arterial tonometry and constitute the analytic cohort [paired augmentation index at 75 bpm (AI@75) *n* = 71; paired RHI *n* = 70; and donor sex known *n* = 70]. Their living donors were assessed before donation with the same device and protocol (*n* = 46; 38 on the same day as the recipient), providing a sex-specific healthy reference. The study was approved by the Swedish Ethical Review Authority (2008/1748-31/2; amendments 2015/1789-32), conducted in accordance with the Declaration of Helsinki; all participants provided written informed consent.

### Clinical and laboratory data

Assessments were performed at baseline (within 1 week before transplantation) and repeated at a median of 24.6 months after transplantation (interquartile range [IQR] 24.0–25.4; range 9.7–30.9; 70 of 71 recipients were reassessed between 18 and 31 months). Anthropometry, resting blood pressure (mean of three seated readings), primary kidney disease, comorbidities, concurrent medications, and donor age and sex (70 of 71 recipients) were obtained from records and patient interview, together with dialysis status, modality and vintage, arteriovenous fistula, and cigarette smoking and snus use at both time points. Fasting baseline samples were assayed at the accredited Karolinska University Laboratory (Table 1). Gut-derived uremic toxins and tryptophan metabolites were quantified by liquid chromatography–tandem mass spectrometry (Supplementary material) in a subset of 29 recipients (22 men, 7 women), as sample availability permitted.

**Table 1:** Baseline characteristics of the study cohort, stratified by recipient sex.

Characteristic	Overall (*n* = 71)	Male (*n* = 48)	Female (*n* = 23)	*P*
Demographic and clinical				
Age, years	48.5 ± 13.6	46.7 ± 13.8	52.2 ± 12.7	.105
Body-mass index, kg/m²	25.2 ± 4.0	25.1 ± 4.0	25.2 ± 4.3	.928
Systolic blood pressure, mmHg	144 ± 18	146 ± 19	141 ± 18	.314
Diastolic blood pressure, mmHg	89 ± 12	90 ± 13	86 ± 10	.117
Mean arterial pressure, mmHg	107 ± 13	109 ± 14	104 ± 11	.146
Heart rate, bpm (*n* = 69)	66 ± 9	65 ± 8	67 ± 11	.409
Kidney replacement and lifestyle				
Pre-emptive transplantation (*n* = 70)	34 (49)	22 (46)	12 (55)	.609
Arteriovenous fistula (*n* = 70)	5 (7)	3 (6)	2 (9)	.646
Current smoking	3 (4)	1 (2)	2 (9)	.243
Snus use	6 (8)	6 (12)	0 (0)	.167
Donor				
Male donor (*n* = 70)	32 (46)	20 (43)	12 (52)	.610
Donor age, years (*n* = 70)	49.7 ± 10.0	50.1 ± 10.3	48.8 ± 9.4	.600
Baseline laboratory				
Creatinine, µmol/l	644 [518–795]	712 [551–856]	541 [457–684]	.007
hs-CRP, mg/l	1.4 [0.7–3.2]	1.5 [0.7–2.7]	0.9 [0.6–4.0]	.811
Albumin, g/l	35 ± 4	35 ± 4	35 ± 4	.764
Calcium, mmol/l	2.32 ± 0.18	2.31 ± 0.19	2.34 ± 0.15	.507
Phosphate, mmol/l	1.70 [1.50–1.95]	1.75 [1.50–2.10]	1.60 [1.45–1.80]	.243
Parathyroid hormone, pmol/l (*n* = 69)	24 [17–40]	24 [19–30]	30 [15–48]	.411
hs-Troponin T, ng/l (*n* = 70)	28 [20–44]	32 [23–52]	20 [15–26]	<.001
Total cholesterol, mmol/l	4.6 ± 1.3	4.3 ± 1.2	5.1 ± 1.2	.018
HDL cholesterol, mmol/l	1.40 [1.10–1.80]	1.25 [1.00–1.40]	1.70 [1.30–2.05]	.001
Triglycerides, mmol/l	1.4 [1.0–1.9]	1.5 [1.2–1.8]	1.3 [1.0–2.0]	.479
Apolipoprotein B, g/l (*n* = 70)	0.84 ± 0.29	0.81 ± 0.30	0.90 ± 0.28	.212
Lipoprotein(a), mg/l (*n* = 67)	30 [10–84]	31 [10–86]	27 [6–81]	.520
HbA1c, mmol/mol (*n* = 69)	34 [31–37]	33 [31–36]	34 [31–38]	.549
Homocysteine, µmol/l	36 [27–46]	36 [28–46]	38 [26–44]	.917
25-OH vitamin D, nmol/l	64 ± 27	61 ± 28	69 ± 25	.228
Interleukin-6, pg/ml (*n* = 47)	4.4 [2.2–6.2]	4.1 [2.1–6.5]	4.5 [2.9–5.4]	.759
Baseline medications				
ACE inhibitor/ARB	38 (54)	22 (46)	16 (70)	.078
Calcium-channel blocker	50 (70)	37 (77)	13 (57)	.098
β-Blocker	44 (62)	31 (65)	13 (57)	.604
Statin	26 (37)	19 (40)	7 (30)	.600
Baseline vascular function				
Reactive hyperemia index	2.44 [1.96–2.88]	2.48 [2.09–2.91]	2.27 [1.84–2.74]	.233
lnRHI	0.88 ± 0.28	0.91 ± 0.29	0.82 ± 0.25	.188
Augmentation index @75, %	4.9 ± 17.4	1.8 ± 14.8	11.4 ± 20.6	.053
Follow-up				
Interval, months	24.6 [24.0–25.4]	24.8 [24.0–25.4]	24.5 [24.0–25.5]	.735
Creatinine, µmol/l	114 [104–138]	121 [109–142]	90 [79–114]	.001

Note: Data are mean ± SD or median [IQR] according to distribution (Shapiro–Wilk test); categorical variables are *n* (%). *P* values: Welch *t*-test or Mann–Whitney *U* test for continuous variables and Fisher exact test for categorical variables, male versus female. Where an *n* is given it denotes the number of participants with available data. Of the 36 recipients receiving dialysis, 14 received hemodialysis and 22 peritoneal dialysis; dialysis vintage was 0.2 [0.0–0.8] years (*n* = 29).

Abbreviations: ACE, angiotensin-converting enzyme; ARB, angiotensin-receptor blocker; HDL, high-density lipoprotein; hs-CRP, high-sensitivity C-reactive protein; lnRHI, natural logarithm of the reactive hyperemia index.

### Peripheral arterial tonometry (EndoPAT)

Microvascular endothelial function and arterial stiffness were assessed using the EndoPAT 2000 (Itamar Medical Ltd, Caesarea, Israel), a validated, operator-independent device [12, 13], after an overnight fast and abstinence from caffeine, tobacco, and vasoactive medications (Supplementary material). The RHI, the ratio of post- to pre-occlusion peripheral arterial tone normalized to the control arm [12], reflects endothelium-dependent, in part NO-mediated vasodilation; because it was right-skewed, it was analyzed throughout as lnRHI, the form validated against cardiovascular outcomes [36]. Native RHI values are reported for description and for the conventional dysfunction threshold of 1.67; all inferential analyses use lnRHI. The AI@75 indexes arterial stiffness, higher values denoting greater stiffness [37], representing the proportion of central pulse pressure attributable to wave reflection and standardized to 75 bpm to account for the heart-rate dependence of reflection timing [38]. Donors underwent an identical protocol, on the same device.

### *Ex vivo* wire myography

In recipients donating tissue at transplantation, a small resistance artery from subcutaneous fat (lumen diameter median 175 µm, IQR 116–245 µm) was mounted on a wire myograph and normalized to optimal internal circumference [39] (Supplementary material). After norepinephrine pre-constriction, endothelium-dependent relaxation to cumulative bradykinin (10^−11^–10^−6^ M) was assessed without and with the NO-synthase inhibitor Nω-nitro-L-arginine methyl ester (L-NAME), the L-NAME-inhibitable and L-NAME-resistant fractions defining the NO and EDH components (percentage of total relaxation) [8]. Endothelium-independent relaxation used sodium nitroprusside, and passive stiffness was indexed by the exponential constant *B* of the passive length–tension relationship (higher *B* denoting stiffer vessels). Bradykinin concentration-response data were available in 37 recipients (23 men, 14 women), the L-NAME protocol in 35, and the passive length–tension relationship in 26 (16 men, 10 women). As vessels were harvested at transplantation, *ex vivo* measures represent the baseline, pre-transplant phenotype. The same protocol was applied to subcutaneous resistance arteries from 46 living donors of the same program (20 men, 26 women; L-NAME protocol in 45), studied at nephrectomy, providing a healthy comparison group. Six of these donors also contributed to the tonometric reference.

#### Immunosuppression, exposures, and outcomes

Standard immunosuppression comprised basiliximab induction and maintenance tacrolimus, mycophenolate mofetil, and tapered corticosteroids. Maintenance regimen was recorded early after transplantation and at follow-up, and tacrolimus trough concentrations at initiation and at approximately 3, 6, 12, and 24 months. Cumulative exposure was summarized as the mean of available troughs. Donor–recipient matching was prespecified as sex-matched (F→F, M→M) or sex-mismatched (F→M, M→F).

#### Sex-specific reference values and standardization

Because both tonometric indices differ between healthy men and women, recipient values were also expressed as *z*-scores against sex-specific reference distributions derived from the living donors (Table 2), a zero denoting the mean of healthy donors of the same sex [14]. Raw values are reported alongside. Male and female reference standard deviations differ, so equal raw deviations yield unequal *z*-scores. Donors were assessed only once, before donation.

**Table 2:** Living kidney donor reference cohort and sex-specific reference values.

Characteristic	All donors (*n* = 46)	Men (*n* = 22)	Women (*n* = 24)	*P*
Characteristics				
Age, years	49.0 ± 9.5	46.9 ± 9.1	51.0 ± 9.5	.140
Heart rate, bpm	59.6 ± 8.4	57.5 ± 7.5	61.6 ± 8.9	.100
Vascular measures				
Reactive hyperemia index	2.47 ± 0.66	2.23 ± 0.40	2.69 ± 0.78	.015
lnRHI	0.876 ± 0.236	0.787 ± 0.183	0.958 ± 0.253	.012
Augmentation index @75, %	−0.8 ± 15.6	−11.7 ± 9.9	9.1 ± 13.1	<.001
RHI distribution				
5th percentile	—	1.60	1.86	
25th percentile	—	2.01	2.25	
Median	—	2.25	2.52	
75th percentile	—	2.40	2.85	
Range	—	1.49–3.10	1.67–5.43	
RHI < 1.67, *n*	—	2	0	

Note: Values are mean ± SD; *P* by Welch *t*-test. Reference distributions used to standardize recipient values were lnRHI 0.787 ± 0.183 (men) and 0.958 ± 0.253 (women), and AI@75 −11.7% ± 9.9% (men) and 9.1% ± 13.1% (women). Recipients with and without a paired donor assessment did not differ in age (46.8 ± 12.8 vs 51.6 ± 14.8 years; *P* = .18), baseline lnRHI (*P* = .88), baseline AI@75 (*P* = .62) or male sex (Fisher *P* = 1.00).

Abbreviations: AI@75, augmentation index at 75 bpm; lnRHI, natural logarithm of the reactive hyperemia index; RHI, reactive hyperemia index.

### Statistical analysis

Continuous variables are summarized as mean ± SD or median (IQR) as appropriate, categorical variables as *n* (%); within-subject changes were tested by paired *t*-test, with Wilcoxon signed-rank tests given alongside in Table S1. The primary endpoints were the changes in lnRHI and AI@75; all other analyses, including the sex × time interaction, matching status, and the four donor→recipient combinations, were exploratory. Sex-dependent trajectories were modeled by linear mixed-effects models with a random participant intercept, fitted by maximum likelihood, the time × sex term testing divergence; all available observations were included, one recipient contributing a baseline measurement only. The lnRHI interaction was re-estimated across a range of specifications: adjustment for age, blood pressure, and body-mass index; for statin use and lipids; for baseline tobacco use; for pre-emptive transplantation; for cumulative tacrolimus exposure; for graft function at follow-up; for donor age and sex; restriction to follow-up ≥18 months; the exact follow-up interval as a continuous time metric; and adjustment for baseline lnRHI (analysis of covariance). Influence of individual participants was assessed by leave-one-out re-estimation. Recipient values were also compared with donors of the same sex by two-sample *t*-test, with 95% confidence intervals (CIs) obtained by bootstrap resampling of both groups; *z*-scores are reported as the descriptive scale. Matched and mismatched recipients were compared by Mann–Whitney *U* test (Cohen’s *d* with bootstrap 95% CI), and ΔAI@75 modeled by ordinary least squares on baseline AI@75, age, sex, and matching status to separate matching from regression to the mean [40]. ΔlnRHI was modeled in the same way, on baseline lnRHI, age, and sex, so that both endpoints were adjusted identically. The four donor→recipient groups were compared by Kruskal–Wallis test. *Ex vivo* comparisons used mixed-effects models (bradykinin concentration as a categorical fixed effect, random vessel intercept) and Mann–Whitney *U* tests, with differences reported as 95% CIs and the smallest difference detectable at 80% power (*α* = 0.05). Sex was compared within recipients and within donors, and a group × sex term tested whether the sex effect varied between groups. Baseline analytes and uremic toxins were screened against ΔlnRHI by Spearman correlation, and discrimination for follow-up endothelial dysfunction (RHI < 1.67) by the area under the receiver-operating-characteristic curve (AUC). *P*-values are reported unadjusted for multiplicity [41], with Benjamini–Hochberg-corrected values alongside in Table S2; full details are in Supplementary material. Tests were two-sided (*α* = 0.05). Analyses were performed in Python 3.12.3 (pandas, numpy, scipy, statsmodels).

## RESULTS

### Study population

Between January 2017 and March 2024, 71 first living-donor kidney transplant recipients completed paired vascular assessments (RHI for 70, AI@75 for 71). The cohort was 68% male (48 men, 23 women), mean age 48.5 ± 13.6 years (Table 1). A total of 34 out of 70 (49%) were transplanted pre-emptively; 5 had an arteriovenous fistula, and current smoking (3 of 71) and snus use (6 of 71, all men) were uncommon and similar between sexes. Comorbidity and primary renal diagnosis were recorded in 28 recipients, among whom hypertension was near-universal (25 of 28) and the leading causes of kidney failure were autosomal dominant polycystic kidney disease (43%), glomerulonephritis (25%), and congenital anomalies of the kidney and urinary tract (11%). Donor sex was known for 70 recipients. Thirty-one transplants were sex-matched (F→F 11, M→M 20) and 39 sex-mismatched (F→M 27, M→F 12). Median follow-up was 24.6 months (IQR 24.0–25.4; range 9.7–30.9), with 70 of 71 recipients reassessed between 18 and 31 months. Tonometry was also available from 46 living donors (22 men, 24 women; mean age 49.0 ± 9.5 years), providing the sex-specific reference used below (Table 2).

### Healthy sex-specific reference values

Healthy male and female donors differed in both vascular measures. Men had lower microvascular reactivity than women (RHI 2.23 ± 0.40 vs 2.69 ± 0.78, *P* = .015; lnRHI 0.787 ± 0.183 vs 0.958 ± 0.253, *P* = .012) and lower wave reflection (AI@75 −11.7 ± 9.9% vs 9.1 ± 13.1%; *P* < .001). Two of 22 healthy men fell below the conventional dysfunction threshold of RHI <1.67, and no healthy woman did, the lowest female value being exactly 1.67 (Table 2). Recipient values were therefore interpreted against sex-specific reference distributions.

### Overall vascular changes

Large-artery stiffness (AI@75) and microvascular function (lnRHI) changed in opposite directions (Fig. 1A and B). AI@75 improved, falling from 4.9% ± 17.4% to −0.8%± 17.2% (Δ −5.7%; 95% CI −9.4 to −2.0; *P* = .003), whereas lnRHI declined marginally from 0.89 ± 0.27 to 0.80 ± 0.28 (ΔlnRHI −0.084; 95% CI −0.167 to −0.001; *P* = .048; native RHI 2.52 → 2.32, *P* = .053). Within individuals the two changes were only modestly correlated (*ρ* = +0.30, *P* = .012).

**Figure 1:**
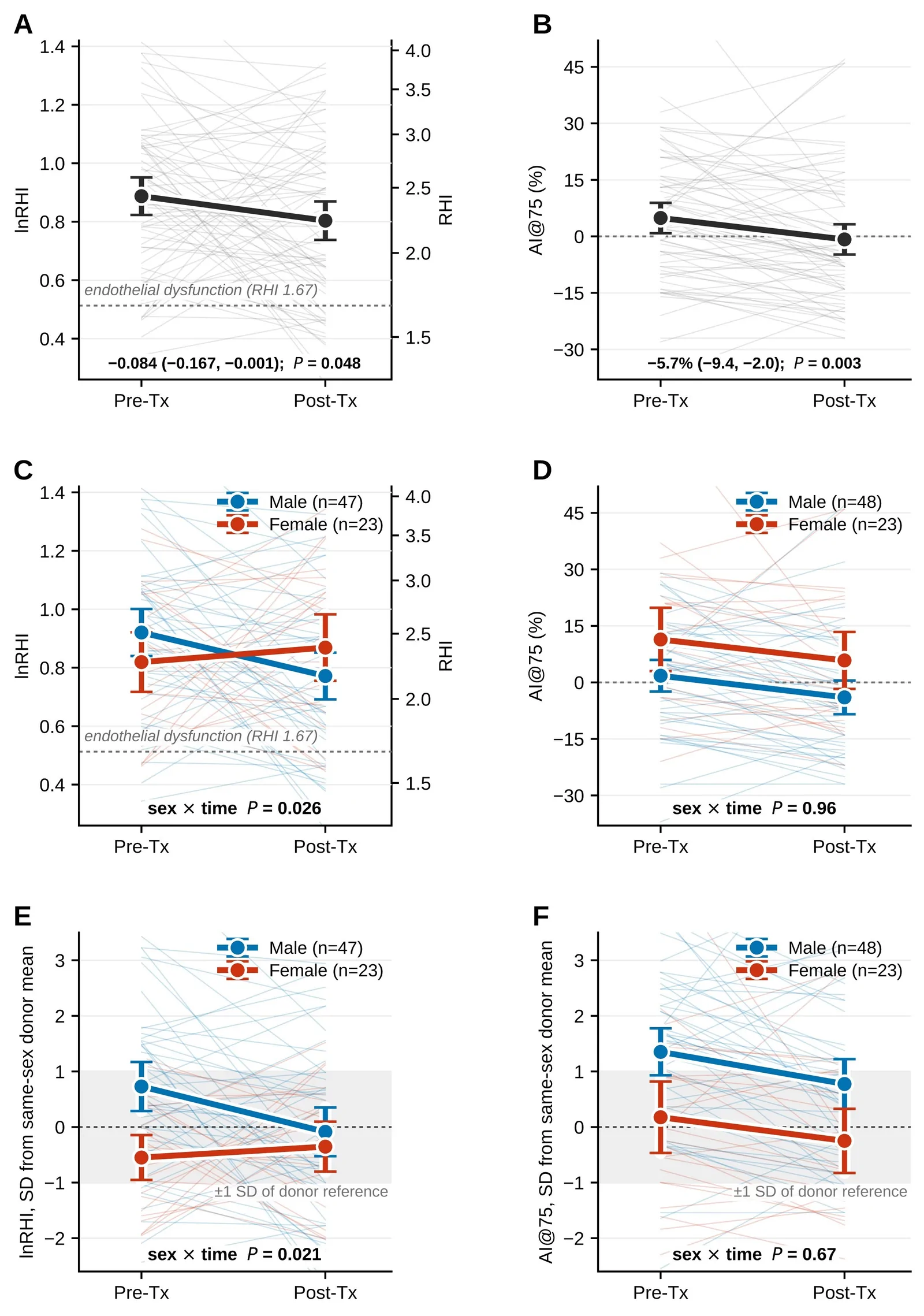
Vascular trajectories before and after living-donor kidney transplantation. (A, B) Whole cohort; (C, D) by recipient sex; (E, F) standardized to the sex-specific living-donor reference. (A, C, E) Microvascular endothelial function as lnRHI, with native RHI on the secondary axis; (B, D, F) AI@75. Dotted lines mark the RHI threshold for endothelial dysfunction (1.67) in panels (A) and (C), and the healthy donor mean in panels (E) and (F), where shading spans ±1 SD. *n* = 70 for RHI and 71 for AI@75.

### Sex-stratified trajectories of vascular function

#### Microvascular function

Trajectories differed by recipient sex (sex × time interaction *β* = −0.190, SE = 0.085, 95% CI −0.357 to −0.022; *P* = .026; Table 3 and Fig. 1C): lnRHI declined in men (0.92 ± 0.28 → 0.77 ± 0.28; Δ −0.149, 95% CI −0.241 to −0.057; *P* = .002) but was unchanged in women (0.82 ± 0.25 → 0.87 ± 0.28; Δ +0.050, 95% CI −0.117 to +0.217; *P* = .54). The between-sex difference in change was −0.199 (bootstrap 95% CI −0.374 to −0.020).

**Table 3:** Sex-stratified vascular trajectories before and after transplantation.

Parameter	Men (*n* = 47/48)			Women (*n* = 23)			Sex × time *β* (*P*)
	Pre-Tx	Post-Tx	Δ (*P*)	Pre-Tx	Post-Tx	Δ (*P*)	
lnRHI	0.92 ± 0.28	0.77 ± 0.28	−0.15 (.002)	0.82 ± 0.25	0.87 ± 0.28	+0.05 (.544)	−0.19 (.026)
RHI	2.61 ± 0.78	2.25 ± 0.64	−0.36 (.003)	2.34 ± 0.59	2.47 ± 0.67	+0.13 (.483)	−0.48 (.020)
AI@75 (%)	1.8 ± 14.8	−4.0 ± 15.8	−5.7 (.007)	11.4 ± 20.6	5.8 ± 18.5	−5.6 (.176)	−0.2 (.963)
lnRHI, *z* ^a^	0.73 ± 1.54	−0.09 ± 1.53	−0.82 (.002)	−0.55 ± 0.99	−0.35 ± 1.10	+0.20 (.544)	−0.97 (.021)
AI@75, *z* ^a^	1.35 ± 1.49	0.77 ± 1.59	−0.58 (.007)	0.18 ± 1.57	−0.25 ± 1.41	−0.42 (.176)	−0.15 (.668)

Note: Values are mean ± SD. Δ is the within-group change with its paired *t*-test *P* value.

Abbreviations: AI@75, augmentation index at 75 bpm; lnRHI, natural logarithm of the reactive hyperemia index; Post-Tx, after transplantation; Pre-Tx, before transplantation; RHI, reactive hyperemia index.

^a^Values standardized against sex-specific living-donor reference distributions (Table 2) and expressed as standard deviations from the mean of donors of the same sex. Because male and female reference standard deviations differ, standardized estimates are not numerically comparable with unstandardized ones. n = 47 men for lnRHI and RHI and 48 for AI@75.

#### Arterial stiffness

AI@75 decreased similarly in both sexes (men −5.7%, 95% CI −9.8 to −1.7, *P* = .007; women −5.6%, 95% CI −13.8 to +2.7, *P* = .18; interaction P = .96; Fig. 1D). Women declined from a higher baseline stiffness (*β* = −9.6%, *P* = .022) that persisted throughout follow-up.

### Recipients relative to healthy donors

Standardized against donors of the same sex, the two compartments behaved differently (Tables 2 and 3 and Fig. 1E and F). Before transplantation, male recipients had higher microvascular reactivity than healthy men (*z* = +0.73; 95% CI +0.15 to +1.47; P = .022), whereas female recipients started below the female reference (z = −0.55; 95% CI −1.14 to +0.01). At follow-up, male recipients did not differ from healthy men (*z* = −0.09; 95% CI −0.79 to +0.56; *P* = .78), while female recipients remained below the female reference (*z* = −0.35; 95% CI −0.97 to +0.29). The sex × time interaction persisted on the standardized scale (*β* = −0.966; 95% CI −1.786 to −0.147; *P* = .021). Arterial stiffness followed a different course, with no divergence by sex (P = .67). Male recipients were stiffer than healthy men before transplantation (*z* = +1.35; 95% CI +0.73 to +2.36; *P* < .001) and remained so afterwards despite improvement (*z* = +0.77; 95% CI +0.16 to +1.60; *P* = .016). Female recipients did not differ from healthy women at either time point (*z* = +0.18 → *z* = −0.25). After transplantation, therefore, arterial stiffness in men was the only measure that remained abnormal relative to same-sex healthy donors.

### Effect of donor–recipient sex matching

In unadjusted analyses, sex-matched recipients showed greater AI@75 improvement than mismatched (−10.8% ± 16.5% vs −1.4% ± 14.1%; *P* = .020; Cohen’s *d* = −0.61, 95% CI −1.04 to −0.20; Fig. 2A), and across the four combinations AI@75 improvement followed a non-significant gradient (F→F −12.8%, M→M −9.7%, F→M −2.6%, M→F +1.1%; Kruskal–Wallis *P* = .11; Fig. 2B). Matching had no effect on microvascular function (ΔlnRHI −0.069 vs −0.078; *P* = .96; *n* = 69; Fig. 2C). Here, the microvascular changes segregated by recipient rather than donor sex (ΔlnRHI F→F +0.01, M→F +0.09, F→M −0.16, M→M −0.11; *P* = .23; Fig. 2D): grouped by donor sex the changes were −0.04 for male and −0.11 for female donors, whereas grouped by recipient sex they were −0.14 for men and +0.05 for women. The apparent stiffness advantage of sex-matched transplantation is attributable to baseline imbalance together with age, with no independent contribution of matching. Matched recipients began far stiffer (baseline AI@75 +10.6% vs +0.5%), and higher baseline stiffness strongly predicted its own subsequent fall (slope −0.42; *r* = −0.46; *P* < .001; Fig. 3A), the high-baseline quartile being predominantly female-donor grafts (12 of 18). When ΔAI@75 was modeled on baseline AI@75, age, sex, and matching status (*R*² = 0.44), only baseline stiffness (*β* = −0.52, *P* < .001) and age (*β* = +0.52, *P* < .001) predicted change; matching contributed nothing independent (*β* = +3.1%, *P* = .32).

**Figure 2:**
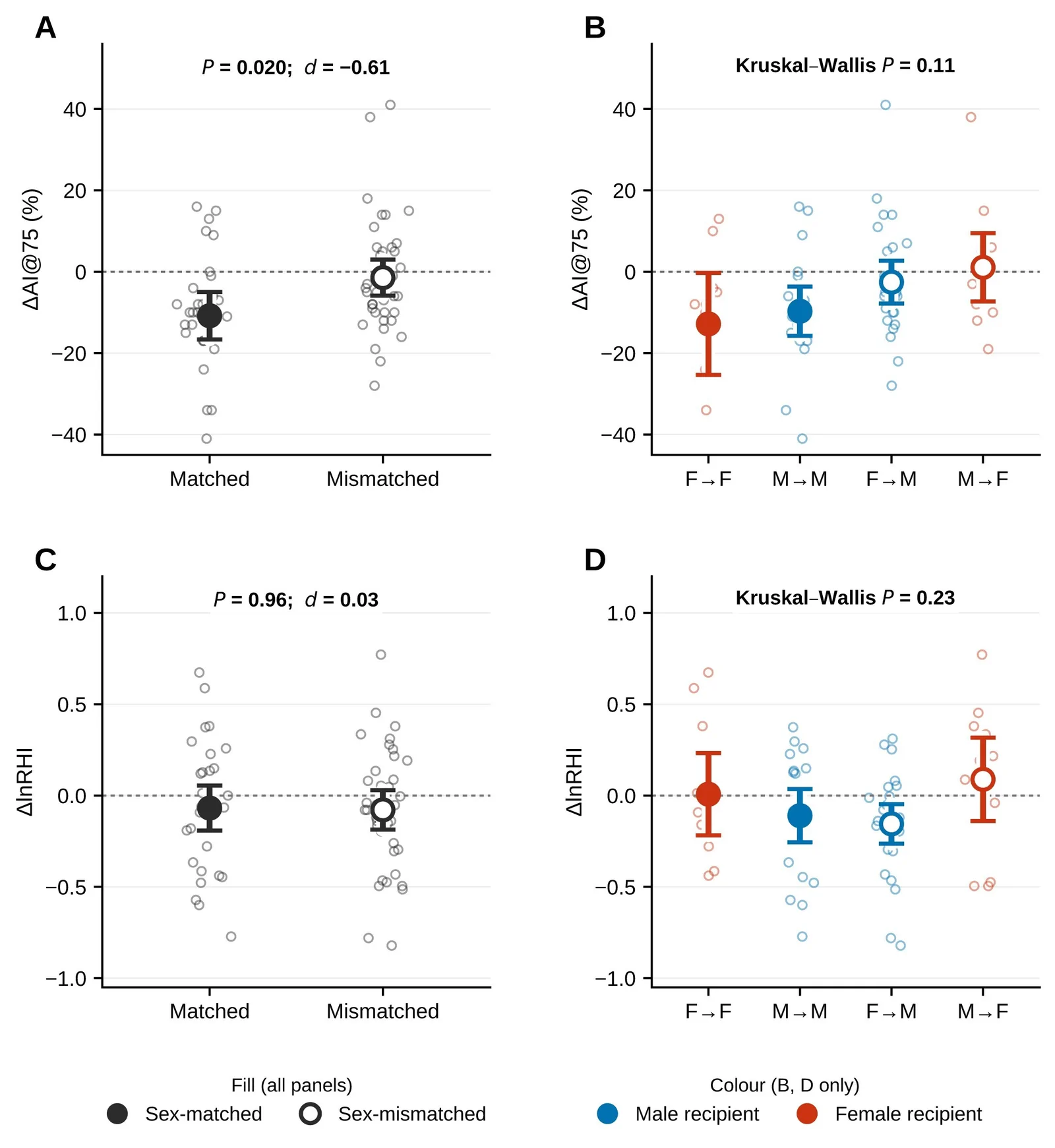
Donor–recipient sex matching and vascular change. Change scores (Post-Tx − Pre-Tx) for AI@75 (A, B) and lnRHI (C, D), by matching status (A, C) and across the four donor→recipient sex combinations (B, D). *n* = 70 for AI@75 and 69 for lnRHI (donor sex known; one male without follow-up measurement).

**Figure 3:**
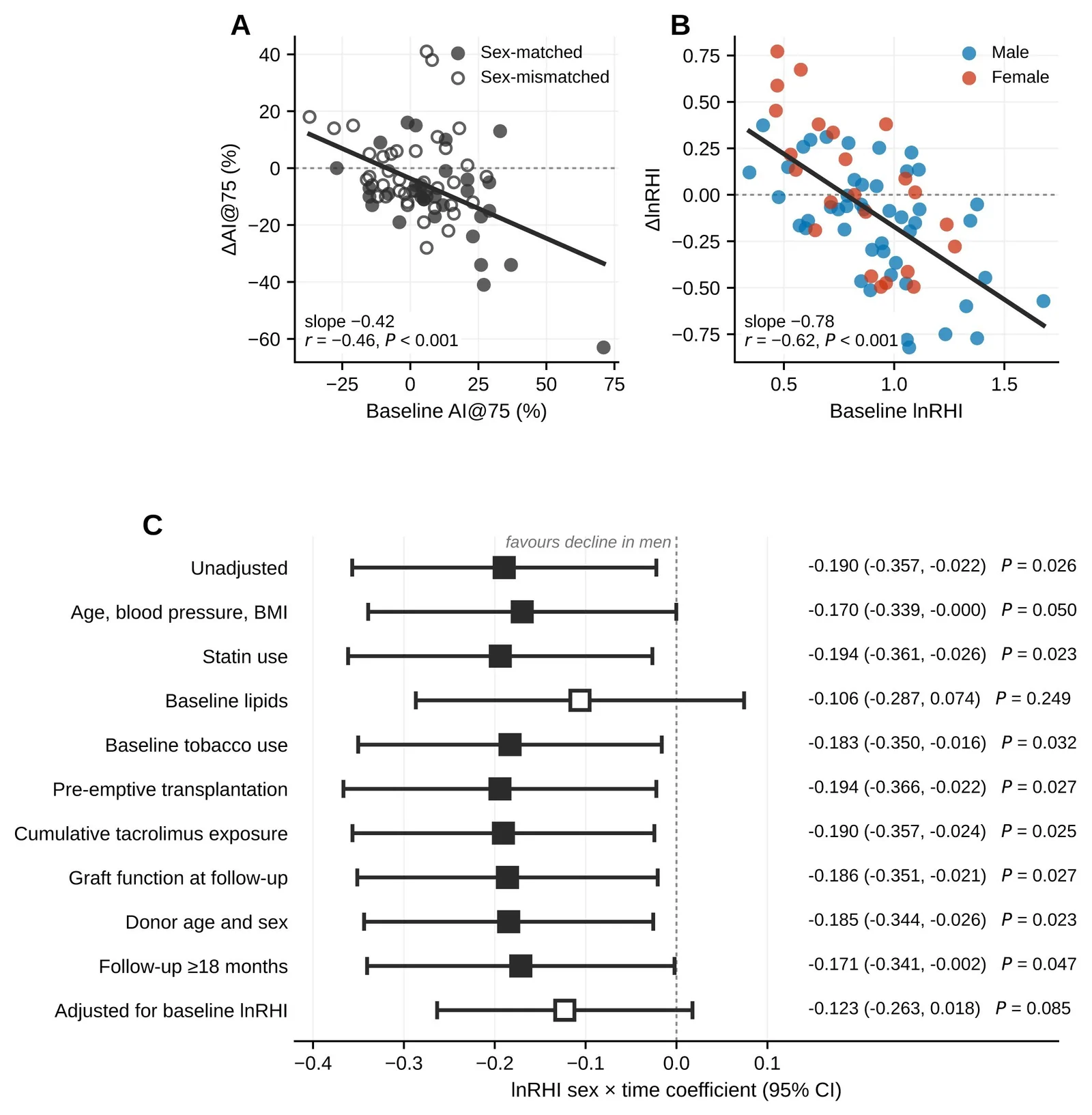
Regression to the mean and robustness of the sex × time interaction. (A, B) Baseline value against subsequent change for AI@75 (A) and lnRHI (B); solid lines are least-squares fits, the dotted line marks no change; symbols indicate matching status (A) and recipient sex (B). (C) lnRHI sex × time coefficient with 95% CI across model specifications; filled squares indicate *P* < .05. *n* = 71 for AI@75 and 70 for lnRHI.

### Sensitivity analyses

Baseline values strongly predicted their own subsequent change for both endpoints (AI@75 *r* = −0.46, Fig. 3A; lnRHI *r* = −0.62, *P* < .001, Fig. 3B). The sex × time interaction was stable in direction across eleven specifications, including adjustment for age, blood pressure, body mass index, statin use, tobacco use, pre-emptive transplantation, tacrolimus exposure, graft function, and donor characteristics (Fig. 3C and Table S2). It was attenuated by adjustment for baseline lnRHI (−0.123; 95% CI −0.263 to +0.018; *P* = .085) and for baseline lipids (−0.106; 95% CI −0.287 to +0.074; *P* = .249). The latter reflects high-density lipoprotein (HDL) cholesterol, which is higher in women (median 1.70 vs 1.25 mmol/l, *P* = .001) and correlated with change in women (*ρ* = +0.53) but not in men (*ρ* = −0.04). Leave-one-out re-estimation moved the unadjusted interaction between −0.215 and −0.157. No baseline analyte predicted ΔlnRHI across the 16 measured (smallest uncorrected *P* = .050, total cholesterol; none surviving correction; Table S3); C-reactive protein (*ρ* = +0.04) and interleukin-6 (*ρ* = +0.06) were unrelated, and the sex differences in baseline biochemistry (Table 1) did not account for it. A uremic-toxin and tryptophan-metabolite panel, measured in the 29 recipients with sufficient stored plasma (22 men, 7 women), showed no association with ΔlnRHI (smallest *P* = .13, none surviving correction; Table S4). Baseline indoxyl sulphate correlated with the follow-up RHI value (*ρ* = −0.43, *P* = .019) but did not differ between the sexes (*P* = 1.00). Baseline lnRHI discriminated poorly for endothelial dysfunction at follow-up (RHI < 1.67; 14 of 70 recipients; AUC 0.61).

### Immunosuppression exposure

All recipients received tacrolimus and prednisolone, and 70 of 71 mycophenolate; at follow-up, 70 of 71 remained on tacrolimus, 66 on mycophenolate and all on prednisolone, with 5 converted to everolimus (Table 4). Tacrolimus exposure did not differ by recipient sex at initiation (9.6 ± 3.4 vs 10.5 ± 5.8 ng/ml; *P* = .46), across the trough series (mean 6.7 ± 1.0 vs 6.7 ± 1.7; *P* = .97), or at 2 years (5.3 ± 1.2 vs 5.0 ± 1.0; *P* = .41; *n* = 53), and was unrelated to change in either endpoint (ΔlnRHI *ρ* = +0.10, *P* = .40; ΔAI@75 *ρ* = +0.02). Adjustment for cumulative exposure left the sex × time interaction unchanged (−0.190; *P* = .025; Fig. 3C).

**Table 4:** Maintenance immunosuppression and tacrolimus exposure, by recipient sex.

	Overall (*n* = 71)	Men (*n* = 48)	Women (*n* = 23)	*P*
Regimen early after transplantation, *n* (%)				
Tacrolimus	71 (100)	48 (100)	23 (100)	1.000
Mycophenolate	70 (99)	47 (98)	23 (100)	1.000
Prednisolone	71 (100)	48 (100)	23 (100)	1.000
Everolimus	2 (3)	2 (4)	0 (0)	1.000
Regimen at follow-up, *n* (%)				
Tacrolimus	70 (99)	47 (98)	23 (100)	1.000
Mycophenolate	66 (93)	45 (94)	21 (91)	.656
Prednisolone	71 (100)	48 (100)	23 (100)	1.000
Everolimus	5 (7)	4 (8)	1 (4)	1.000
Cyclosporine	1 (1)	1 (2)	0 (0)	1.000
Azathioprine	1 (1)	0 (0)	1 (4)	.324
Tacrolimus trough concentration, ng/ml				
At initiation	9.9 ± 4.3	9.6 ± 3.4	10.5 ± 5.8	.458
≈3 months	6.8 ± 1.4	7.0 ± 1.3	6.6 ± 1.7	.301
≈6 months	5.6 ± 1.2	5.6 ± 0.9	5.8 ± 1.7	.542
≈12 months (*n* = 65)	5.5 ± 1.3	5.6 ± 1.4	5.2 ± 1.2	.280
≈24 months (*n* = 53)	5.2 ± 1.1	5.3 ± 1.2	5.0 ± 1.0	.409
Mean of available troughs	6.7 ± 1.2	6.7 ± 1.0	6.7 ± 1.7	.969

Note: Values are *n* (%) or mean ± SD. *P* by Fisher exact test (categorical) or Welch *t*-test (continuous), male versus female. Where an *n* is given it denotes the number of participants with an available trough concentration; all other variables were complete (*n* = 71).

### Baseline resistance-artery function and structure

Wire myography was available in 37 recipients (23 men, 14 women; L-NAME protocol in 35, passive length–tension relationship in 26) on arteries of median lumen diameter 175 µm (IQR 116–245). Endothelium-dependent relaxation to bradykinin was similar between sexes across the concentration range (sex effect *P* = .83; no concentration × sex interaction, smallest *P* = .59; Fig. 4A); at the maximal concentration the difference was −2.1 percentage points (95% CI −16.5 to +12.4), the study being able to detect a difference of ∼18 percentage points at 80% power. Relaxation was predominantly EDH-mediated (NO component 21.2% ± 24.9% in men vs 11.2% ± 14.0% in women, *P* = .15; EDH component 78.8% vs 88.8%; Fig. 4C). Endothelium-independent relaxation and contractile responses were similar (*P* = .07 and .84). Passive stiffness (constant B, 26 vessels: 16 men, 10 women) was higher in women (14.9 vs 9.9 mm⁻¹, *P* = .048; Fig. 4D), independent of vessel caliber (*ρ* = −0.15). *B* was uncorrelated with *in vivo* AI@75 within individuals (*ρ* = −0.13, *P* = .53) and did not predict either trajectory (ΔlnRHI *ρ* = +0.23, *P* = .26; ΔAI@75 *ρ* = −0.10, *P* = .63). Baseline *ex vivo* relaxation was unrelated to baseline lnRHI (*ρ* = −0.18, *P* = .29) or to its change (*ρ* = −0.04, *P* = .82). No sex difference in resistance-artery function was detectable before transplantation, women’s vessels differing only in being structurally stiffer.

**Figure 4:**
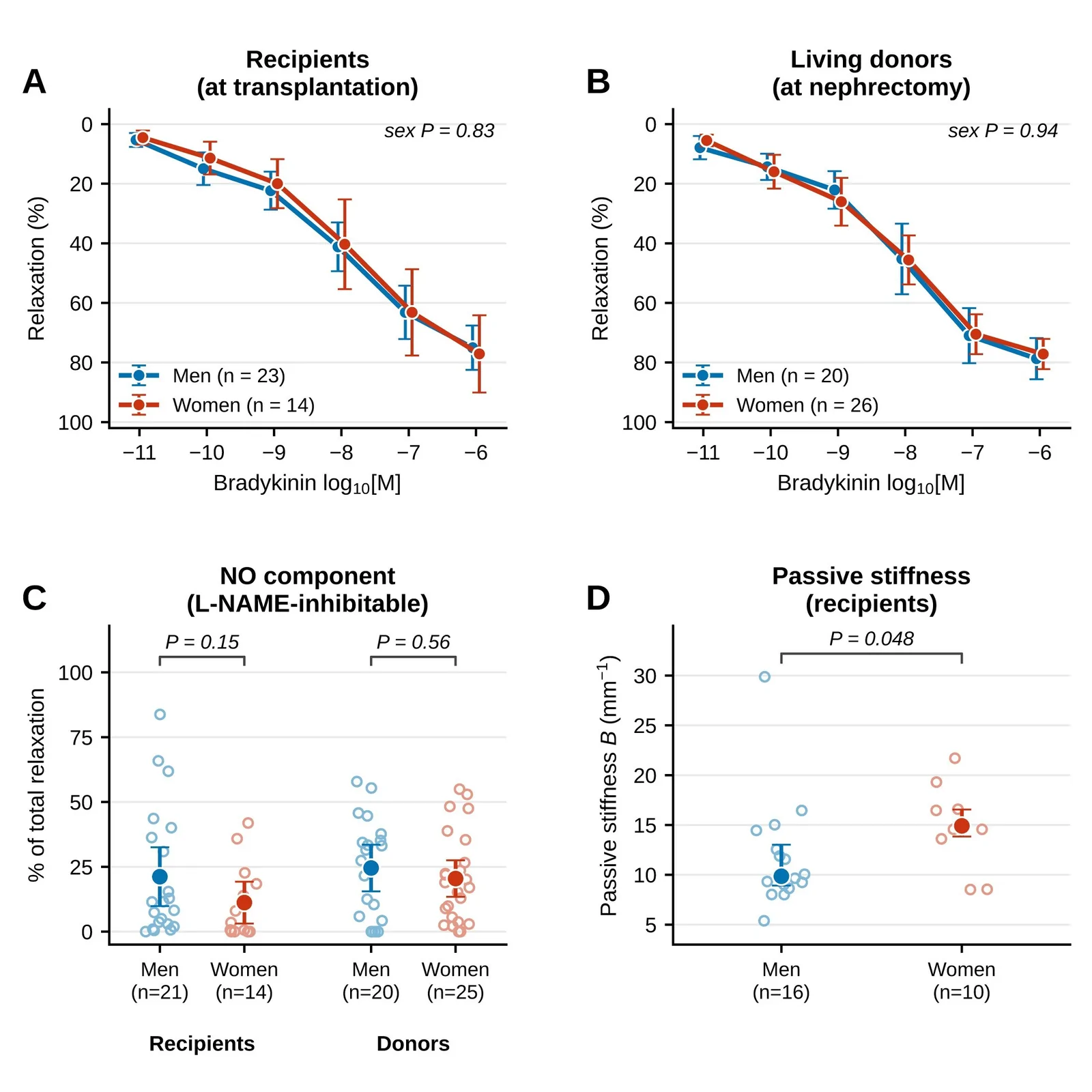
*Ex vivo* resistance-artery function and structure in recipients before transplantation and in healthy living donors. (A, B) Endothelium-dependent relaxation to cumulative bradykinin in recipients at transplantation (A) and in living donors at nephrectomy (B); data are mean ± 95% CI, and *P* is the sex effect from the mixed-effects model. (C) NO component of bradykinin-induced relaxation (L-NAME–inhibitable fraction at 10⁻⁶ M), as a percentage of total relaxation, in recipients and donors; the EDH component is its complement. Small open symbols are individual vessels, bold symbols mean ± 95% CI; *P* by Mann–Whitney *U* test. (D) Passive stiffness in recipients, indexed by the exponential constant *B* of the passive length–tension relationship (*T* = *A*·e^BC^); small open symbols are individual vessels, bold symbols median with interquartile range; *P* by Mann–Whitney *U* test. Men are shown in blue, women in red. Values by concentration and sex are given in Table S5. EDH, endothelium-dependent hyperpolarization; NO, nitric oxide.

Vessels from 46 living donors (20 men, 26 women) of similar age (49.4 ± 11.0 vs 50.4 ± 13.6 years; *P* = .72) provided a healthy comparison. In donors, relaxation to bradykinin did not differ between men and women across the concentration range (sex effect *P* = .94; no concentration × sex interaction, smallest *P* = .16; Fig. 4B). At the maximal concentration it was 78.7% ± 14.8% in men and 77.2% ± 12.6% in women (difference + 1.5 percentage points, 95% CI −6.8 to +9.9), the smallest difference detectable at 80% power being 11 percentage points. Female donors were older than male donors (52.5 ± 10.8 vs 45.3 ± 10.1 years; *P* = .024), and adjustment for age left the sex effect unchanged (*P* = .95). The NO component was similar in male and female donors (24.5% ± 19.2% vs 20.5% ± 17.1%; *P* = .56; Fig. 4C). Values by concentration and sex are given in Table S5. Maximal relaxation to bradykinin did not differ between groups (77.8% ± 13.4% in donors vs 75.8% ± 19.1% in recipients; *P* = .59), nor did relaxation across the concentration range (−3.4 percentage points in recipients, 95% CI −8.8 to +2.0; *P* = .22). The NO and EDH components did not differ between groups (NO 22.3% ± 18.0% vs 17.2% ± 21.5%; *P* = .11), and no group × sex interaction was detected for relaxation or the NO component (*P* = .84 and .51).

## DISCUSSION

In this prospective cohort of living-donor kidney transplant recipients, the two vascular compartments followed different courses over 2 years. Arterial stiffness improved across the cohort, irrespective of recipient sex. Microvascular endothelial function did not: it declined in men and was unchanged in women. The difference was stable in direction across adjusted models but attenuated on adjustment for baseline values and for lipids. Within individuals the two changes were only modestly correlated, indicating largely independent processes rather than a single vascular trajectory. Donor–recipient sex matching, which appeared to favor stiffness regression, conferred no benefit once baseline stiffness was accounted for.

Interpreting these trajectories requires knowing what normal looks like, and for neither measure is this straightforward. The threshold in common use, RHI < 1.67, was derived in patients referred for coronary angiography [12] and adopted in consensus criteria for vascular failure [42], but performs poorly in healthy people, classifying 29.5% of 1812 healthy Swedish adults as having endothelial dysfunction [43]. Nor is it sex-specific. Among our donors, 2 of 22 healthy men fell <1.67 and no healthy woman did, reactive hyperemia being lower in men, consistent with the Framingham Heart Study [14]. Augmentation index poses a different difficulty. It is consistently higher in women [44], but this reflects wave morphology and body size as much as arterial properties, and measures of reflection derived from pressure and flow show no sex difference [45]. We therefore compared recipients with their own donors, assessed on the same device in the same laboratory.

Before transplantation, male recipients had higher reactive hyperemia than healthy men, an unexpected finding for which we have no explanation. At follow-up, they were indistinguishable from them: their microvascular function fell to the level of healthy men, not below it. Arterial stiffness went the other way. Male recipients were much stiffer than healthy men before transplantation, and although they improved, they remained stiffer at follow-up. Female recipients sat below their own reference for reactive hyperemia at both time points, and close to it for stiffness. Male recipients therefore ended indistinguishable from healthy men in microvascular function but remained stiffer than them.

Why the microvascular trajectory differed by sex remains unclear, and none of the exposures we measured accounts for it. Calcineurin inhibitors are the most plausible candidate, impairing endothelial function experimentally through reduced nitric oxide signaling and enhanced Transforming Growth Factor beta (TGF-β) activity [28]. Yet tacrolimus exposure did not differ between the sexes at any point over 2 years, showed no association with change in either measure, and adjustment for cumulative exposure left the sex difference unaltered. The regimen was uniform, so a steroid-sparing or calcineurin-sparing effect could not be examined and equally could not confound. Smoking, snus use, pre-emptive transplantation, and donor characteristics made no difference, and neither 16 baseline analytes nor a panel of gut-derived uremic toxins predicted the decline. Adjustment for lipids did affect the sex difference, but HDL cholesterol is higher in women, so adjusting for it could be considered as partly adjusting for sex. Before transplantation, resistance arteries from men and women showed no detectable difference in relaxation to bradykinin, or in its nitric oxide and EDH components, and neither did those of healthy donors, so the difference does not appear to reside in the vessel itself. That no measured exposure accounts for the fall is consistent with its ending at the healthy male value rather than below it.

Sex-matched transplantation appeared at first to favor stiffness regression, but the advantage disappeared once baseline stiffness was considered: matched recipients simply started stiffer, and stiffer arteries improved more. This matters because donor–recipient sex is thought to influence outcome through mechanisms specific to one direction of mismatch. Female recipients of male kidneys develop antibodies against H-Y minor histocompatibility antigens, which associate with acute rejection [24], while male recipients of female kidneys may be relatively nephron-underdosed, with worse early graft function and higher graft loss [26], though findings across registries conflict. We found no difference between matched and mismatched transplantation once baseline stiffness was accounted for, and the microvascular changes showed no relation to donor sex, segregating instead by recipient sex.

Whether women’s arteries are genuinely stiffer than men’s is difficult to establish from tonometry, since augmentation index depends on wave morphology, stature, and heart rate as well as on the vessel wall, and reflection-based measures show no sex difference [45]. This limits between-sex comparison of augmentation index, though not comparison of recipients with donors of the same sex. Direct mechanical testing of isolated resistance arteries avoids these influences altogether. Women’s vessels were stiffer, independent of caliber, though this rests on 26 vessels. Endothelium-dependent relaxation showed no detectable difference between the sexes in recipients before transplantation or in healthy donors, with differences larger than ∼17 and 10 percentage points, respectively, excluded by the 95% CIs. The same dissociation has been reported in subcutaneous small arteries of comparable caliber in metabolic syndrome, where wall structure predicted cardiac target-organ damage while endothelial function did not [46]. Neither *ex vivo* measure predicted the *in vivo* trajectory, and passive stiffness was uncorrelated with augmentation index within individuals. Relaxation of recipient vessels did not differ detectably from that of vessels from healthy donors of similar age, with a similar reliance on nitric oxide and endothelium-dependent hyperpolarization.

### Clinical implications

Two implications follow. First, a fall in reactive hyperemia after transplantation should not be read as endothelial injury without reference to what is normal for that patient’s sex: the men in this cohort fell to the healthy male value, not below it, and the same reading carries a different meaning in men and women. Second, the abnormality that persisted at follow-up was arterial stiffness in men, not microvascular function in either sex. Whether either trajectory predicts cardiovascular events is untested here, and no surrogate vascular measure has been shown to track the mortality benefit of transplantation itself. The wider outcome literature shows why sex-specific comparison matters: male transplant recipients carry the higher absolute cardiovascular risk, yet women lose more life expectancy relative to women in the general population [47]. Which sex appears worse seems to depend on the comparator chosen.

### Limitations

Several limitations apply. The female sample was small (*n* = 23), and the sex × time interaction attenuated on adjustment for baseline endothelial function and for lipids, though the decline in men did not. The interpretation offered here depends on male recipients having higher reactive hyperemia than healthy men before transplantation, which was unexpected and requires confirmation. The donor reference rests on 22 men and 24 women assessed once. Sex-hormone and menopausal status were not recorded, and primary renal diagnosis was available in 28 of 71 recipients. Augmentation index and RHI derive from the same tonometric signal, and their changes were correlated, so a shared hemodynamic or instrumental determinant cannot be excluded. The *ex vivo* measurements were made once, before transplantation, and cannot show whether vessel properties changed thereafter. No sex difference in resistance-artery relaxation was detectable in recipients or donors, but differences of up to ∼17 and 10 percentage points, respectively, could not be excluded, and cell-autonomous, sex-chromosome-linked differences in endothelial gene expression, and redox metabolism may emerge under stress [48, 49] and therefore would not be captured by a single baseline assay. Finally, this was a single-center, Northern European, exclusively living-donor cohort with no linked cardiovascular outcomes, and the findings may not extend to deceased-donor or more ethnically diverse populations.

### Future directions

Sex-specific reference values for digital tonometry need establishing in larger healthy cohorts than 46 donors. The higher reactive hyperemia of male recipients before transplantation is unexplained and requires confirmation in an independent cohort. Whether residual arterial stiffness in male recipients carries prognostic weight will require linkage to cardiovascular events. Paired pre- and post-transplant myography is feasible because resistance arteries can be sampled by subcutaneous fat biopsy [46] and would then show whether vessel functional and structural properties themselves change.

## CONCLUSIONS

After living-donor kidney transplantation, arterial stiffness improved irrespective of recipient sex, while microvascular endothelial function declined in men and not in women. Against contemporaneous living donors of the same sex, the fall in men ended at the healthy male value, whereas arterial stiffness in men remained above it. What persisted at follow-up was therefore conduit stiffness in men, not microvascular dysfunction in either sex. Nothing measured, including tacrolimus exposure, accounted for the decline in men, and donor–recipient sex matching conferred no benefit once baseline stiffness was considered. These findings are hypothesis-generating and rest on comparison with sex-specific healthy references, which are not available for either measure in routine use.

## Supplementary Material

sfag382_Supplemental_Files

## Data Availability

The data underlying this article will be shared upon reasonable request to the corresponding author.
