# Supplementary material for: Sex dimorphism in vascular trajectories after living-donor kidney transplantation: a prospective exploratory cohort study
Source: Clin Kidney J. 2026 Oct 27;19(11):sfag382. doi: 10.1093/ckj/sfag382 (PMC13760223; doi:10.1093/ckj/sfag382)
Supplement: Supplementary file 1 [file sfag382_supplemental_files.zip › Supplementary_Methods.docx]

# Supplementary Methods

This file provides the detailed measurement protocols summarized in the main-text Materials and Methods.

## S1. Peripheral arterial tonometry (EndoPAT)

Microvascular endothelial function and arterial stiffness were assessed with the EndoPAT 2000 device (Itamar Medical Ltd., Caesarea, Israel), a validated, operator-independent method for noninvasive vascular assessment. Measurements were performed in a temperature-controlled room (22–24 °C) after an overnight fast and abstinence from caffeine, tobacco, and vasoactive medications for at least 12 hours. Participants rested supine for 10 minutes before probe placement. Pneumatic probes were applied to the index fingers of both hands to record pulsatile arterial volume changes (peripheral arterial tone, PAT). Following a 5-minute baseline recording, arterial flow to the test arm was occluded for 5 minutes by inflating a blood-pressure cuff placed on the upper arm to at least 60 mmHg above systolic pressure (minimum 200 mmHg). The contralateral arm served as a control to correct for systemic changes. Post-occlusion recording continued for 5 minutes following cuff deflation.

The following parameters were derived automatically by the EndoPAT software (version 3.4.4):

**Reactive hyperemia index (RHI).** The measure of microvascular endothelial function, calculated as the ratio of post-deflation to pre-occlusion peripheral arterial tone amplitude in the test arm, normalized to the control arm, reflecting endothelium-dependent, nitric-oxide-mediated vasodilation in resistance vessels. Because RHI is right-skewed, microvascular function was analyzed primarily as its natural logarithm (lnRHI), the form validated against cardiovascular risk factors and outcomes.

**Augmentation index normalized to 75 bpm (AI@75).** A measure of arterial stiffness from pulse-wave analysis, representing the proportion of central pulse pressure attributable to wave reflection and standardized to a heart rate of 75 bpm to account for the heart-rate dependence of reflection timing; higher values indicate greater arterial stiffness. Device-exported AI@75 values recorded as proportions were rescaled to percentages so that all observations lay on a common percentage scale.

## S2. Ex vivo wire myography

In recipients donating tissue at transplantation, a small resistance artery from subcutaneous fat (lumen diameter median 175 µm, interquartile range 116–245 µm, derived as internal circumference/π) was studied by wire myography. Segments were mounted on a wire myograph in physiological salt solution at 37 °C, gassed with 95% O₂/5% CO₂, and normalized to their optimal internal circumference; smooth-muscle viability was confirmed with high-potassium solution. Subcutaneous resistance arteries from 46 living donors (20 men, 26 women), obtained at nephrectomy, were studied identically.

After stable pre-constriction with norepinephrine, endothelium-dependent relaxation was assessed by cumulative bradykinin (10⁻¹¹–10⁻⁶ M) in the absence and presence of the nitric-oxide synthase inhibitor L-NAME; the L-NAME-inhibitable fraction defined the nitric-oxide (NO) component and the L-NAME-resistant fraction the endothelium-dependent hyperpolarization (EDH) component, each expressed in absolute terms and as a percentage of total bradykinin-induced relaxation. For comparisons between groups and sexes, the NO component was calculated at 10⁻⁶ M as the difference between relaxation without and with L-NAME, expressed as a percentage of relaxation without L-NAME; it was set to zero where relaxation with L-NAME equaled or exceeded relaxation without it (five recipient and six donor vessels), and the EDH component was taken as its complement. Vessels without a complete L-NAME curve were excluded from these analyses (two recipients, one donor). Endothelium-independent relaxation was assessed with sodium nitroprusside (10⁻⁹–10⁻⁴ M). Relaxation was expressed as the percentage reversal of norepinephrine-induced tone.

Passive stiffness was quantified from the length–tension relationship recorded during normalization, fitted to an exponential function, *T* = *A*·e*^BC^*, where *T* is passive wall tension (mN/mm), *C* is the internal circumference (mm), *A* is a scaling constant (mN/mm), and *B* is the exponential rate constant (mm⁻¹); the constant *B*, indexing the steepness of the curve, was taken as the measure of passive stiffness. Because recipient vessels were harvested at transplantation, ex vivo measurements represent the baseline, pre-transplant vascular phenotype.

## S3. Uremic toxin and tryptophan-metabolite quantification

In a subset of recipients (n = 29), protein-bound uremic toxins (p-cresyl sulphate, phenyl sulphate, indoxyl sulphate, CMPF, hippuric acid, and their glucuronide conjugates), phenylacetylglutamine, and tryptophan-pathway metabolites (tryptophan, kynurenine, kynurenic acid) were quantified in baseline plasma. Total concentrations were measured by ultra-performance liquid chromatography coupled with tandem mass spectrometry (UPLC–MS/MS) after deproteinization and filtration. Trimethylamine-N-oxide was quantified by LC–MS/MS using stable-isotope dilution (TMAO-d9 internal standard).

## S4. Statistical analysis, extended

**Model estimation.** Linear mixed-effects models included a random participant intercept and were fitted by maximum likelihood rather than restricted maximum likelihood; the two estimators give the same point estimates but slightly different standard errors, and results are reported under maximum likelihood throughout. All available observations were included: one recipient completed baseline but not follow-up tonometry for RHI and contributed a baseline measurement only, giving 141 observations from 71 participants for lnRHI and 142 from 71 for AI@75.

**Covariate adjustment.** Baseline covariates are time-invariant and therefore leave a within-person change contrast essentially unaltered when entered as main effects. Adjusted models consequently entered each covariate as a covariate × time interaction, which adjusts the trajectory rather than the level. The prespecified adjustment set was age, mean arterial pressure and body-mass index; additional specifications are listed in Figure 3C. Because a model carrying more interaction terms than the sample supports will attenuate any interaction, estimates are presented across the full range of specifications rather than as a single adjusted value.

**Analysis of change.** Change-score and covariance-adjusted models answer different questions when groups are not randomized: the former asks whether trajectories differ, the latter whether groups differ at follow-up conditional on identical baseline. Both are reported for both endpoints, and neither is treated as arbitrating the other [Vickers AJ, Altman DG. *BMJ* 2001;323:1123–1124, 10.1136/bmj.323.7321.1123].

**Reference standardization.** Sex-specific reference means and standard deviations were computed separately from male and female donors (Table 2). Confidence intervals for standardized means were obtained by bootstrap resampling of both the recipient sample and the donor reference (5000 resamples, seed 20260821), so that uncertainty in the reference is propagated. Recipients and donors were compared by two-sample t-test with unequal variances; one-sample tests of z-scores against zero were not used, because the reference derives from 22 men and 24 women and carries its own sampling error. Because the male and female reference standard deviations differ (0.183 and 0.253 for lnRHI), equal raw deviations yield unequal z-scores between sexes; raw values are therefore reported alongside all standardized values. The female reference was right-skewed and included one donor with a reactive hyperemia index of 5.43; standardization was therefore repeated with that donor excluded (female reference lnRHI 0.926 ± 0.203). Male values were unaffected by definition; the sex × time interaction on the standardized scale was −1.012 (P = 0.024). All donors were retained in the primary analysis, the value lying within the tail of a right-skewed distribution in a screened healthy individual.

**Precision of null comparisons.** For between-sex comparisons of ex vivo measures, the smallest detectable difference was computed as 2.80 × √(1/n₁ + 1/n₂) × pooled SD, corresponding to 80% power at α = 0.05. This prospective precision statement is reported in place of retrospective power calculated from the observed p-value.

**Ex vivo models.** Bradykinin concentration–response curves were analyzed by linear mixed-effects models with concentration as a categorical fixed effect and a random vessel intercept, fitted by maximum likelihood. The sex effect is the sex coefficient (men − women) in the model without interaction terms, a difference assumed constant across concentrations; the concentration × sex interaction was tested by a likelihood-ratio test of the five interaction terms. Recipients and donors were analyzed separately and then jointly, a group × sex term testing whether the sex effect differed between groups. Differences at the maximal concentration (10⁻⁶ M) were estimated with the Welch t-test. The NO component was compared by Mann–Whitney U test, and its group × sex interaction by ordinary least squares. Because female donors were older than male donors, the donor sex effect was also estimated with adjustment for age.

**Multiplicity.** Adjustment for multiple comparisons is required in confirmatory research, where several tests are combined into a single decision [Bender R, Lange S. *J Clin Epidemiol* 2001;54:343–349, 10.1016/s0895-4356(00)00314-0]. The analyses reported here beyond the two primary endpoints are exploratory, and all analyses performed are reported; p-values are therefore presented without adjustment [Rothman KJ. *Epidemiology* 1990;1:43–46, PMID: 2081237; Perneger TV. *BMJ* 1998;316:1236–1238, 10.1136/bmj.316.7139.1236]. Benjamini–Hochberg-corrected values are given alongside uncorrected values in Supplementary Table S2 for reference.

**Exploratory screens.** Baseline analytes (16, listed in Table 1) and the uremic toxin and tryptophan-metabolite panel (15 analytes, n = 29) were screened against ΔlnRHI by Spearman correlation. Discrimination of baseline measures for endothelial dysfunction at follow-up (RHI < 1.67) was assessed by the area under the receiver-operating-characteristic curve.

**Software.** Analyses were performed in Python 3.12.3 with pandas 3.0.2, numpy 2.4.4, scipy 1.17.1 and statsmodels 0.14.6.
