# Supplementary material for: Sex dimorphism in vascular trajectories after living-donor kidney transplantation: a prospective exploratory cohort study
Source: Clin Kidney J. 2026 Oct 27;19(11):sfag382. doi: 10.1093/ckj/sfag382 (PMC13760223; doi:10.1093/ckj/sfag382)
Supplement: Supplementary file 1 [file sfag382_supplemental_files.zip › Supplementary_Tables.docx]

**Supplementary Table S1. Within-group changes: parametric and non-parametric tests.**

| **Endpoint** | **Group** | **n** | **Change** | **Paired t** | **Wilcoxon** |
| --- | --- | --- | --- | --- | --- |
| lnRHI | All | 70 | −0.084 | 0.048 | 0.055 |
| lnRHI | Men | 47 | −0.149 | 0.002 | 0.005 |
| lnRHI | Women | 23 | +0.050 | 0.544 | 0.638 |
| RHI | All | 70 | −0.20 | 0.053 | 0.064 |
| RHI | Men | 47 | −0.36 | 0.003 | 0.007 |
| RHI | Women | 23 | +0.13 | 0.483 | 0.570 |
| AI@75 (%) | All | 71 | −5.7 | 0.003 | 0.001 |
| AI@75 (%) | Men | 48 | −5.7 | 0.007 | 0.005 |
| AI@75 (%) | Women | 23 | −5.6 | 0.176 | 0.152 |

Change is the mean within-group difference (Post-Tx − Pre-Tx). P values from paired t-test and Wilcoxon signed-rank test. Paired t-test values are those reported in the main text.

**Supplementary Table S2. Inventory of all analyses, with prespecification status.**

| **Analysis** | **Status** | **Estimate** | **P** |
| --- | --- | --- | --- |
| Change in lnRHI, whole cohort | Primary | −0.084 | 0.048 |
| Change in AI@75, whole cohort | Primary | −5.7% | 0.003 |
| Sex × time, lnRHI | Exploratory | −0.190 | 0.026 |
| adjusted for age, blood pressure, BMI | Exploratory | −0.170 | 0.050 |
| adjusted for statin use | Exploratory | −0.194 | 0.023 |
| adjusted for baseline lipids | Exploratory | −0.106 | 0.249 |
| adjusted for baseline tobacco use | Exploratory | −0.183 | 0.032 |
| adjusted for pre-emptive transplantation | Exploratory | −0.194 | 0.027 |
| adjusted for cumulative tacrolimus exposure | Exploratory | −0.190 | 0.025 |
| adjusted for graft function at follow-up | Exploratory | −0.186 | 0.027 |
| adjusted for donor age and sex | Exploratory | −0.185 | 0.023 |
| restricted to follow-up ≥18 months | Exploratory | −0.171 | 0.047 |
| continuous follow-up interval (per year)* | Exploratory | −0.086 | 0.039 |
| adjusted for baseline lnRHI | Exploratory | −0.123 | 0.085 |
| Sex × time, AI@75 | Exploratory | −0.2% | 0.963 |
| Sex × time, lnRHI, donor-referenced z | Exploratory | −0.966 | 0.021 |
| Sex × time, AI@75, donor-referenced z | Exploratory | −0.153 | 0.668 |
| Matching, ΔAI@75, unadjusted | Exploratory | −9.4% | 0.020 |
| adjusted for baseline AI@75 | Exploratory | −5.5% | 0.120 |
| adjusted for baseline, age and sex | Exploratory | +3.1% | 0.321 |
| Matching, ΔlnRHI, unadjusted | Exploratory | +0.009 | 0.957 |
| Four donor→recipient groups, ΔAI@75 | Exploratory | Kruskal–Wallis | 0.113 |
| Four donor→recipient groups, ΔlnRHI | Exploratory | Kruskal–Wallis | 0.232 |
| Baseline analyte screen (16 analytes) | Exploratory | smallest ρ = +0.24 | 0.050 |
| Uremic toxin panel (15 analytes, n = 29) | Exploratory | smallest ρ = +0.29 | 0.125 |
| Discrimination for RHI < 1.67 at follow-up | Exploratory | AUC 0.61 | — |
| Ex vivo bradykinin relaxation, sex effect, recipients (men − women) | Exploratory | +0.9 pp | 0.834 |
| at maximal concentration | Exploratory | −2.1 pp | 0.770 |
| Ex vivo bradykinin relaxation, sex effect, donors (men − women) | Exploratory | −0.3 pp | 0.944 |
| at maximal concentration | Exploratory | +1.5 pp | 0.710 |
| adjusted for age | Exploratory | −0.3 pp | 0.948 |
| Ex vivo bradykinin relaxation, recipients − donors | Exploratory | −3.4 pp | 0.222 |
| at maximal concentration | Exploratory | −2.0 pp | 0.588 |
| group × sex | Exploratory | +1.2 pp | 0.835 |
| Ex vivo NO component, sex effect, recipients (men − women) | Exploratory | +10.0 pp | 0.152 |
| Ex vivo NO component, sex effect, donors (men − women) | Exploratory | +4.1 pp | 0.560 |
| Ex vivo NO component, recipients − donors | Exploratory | −5.1 pp | 0.107 |
| group × sex | Exploratory | +5.9 pp | 0.510 |
| Ex vivo passive stiffness, sex effect (women − men) | Exploratory | +5.0 mm⁻¹ | 0.048 |

The primary endpoints were the changes in lnRHI and AI@75. All other analyses were exploratory and P values are unadjusted for multiplicity. Estimates for the sex × time interaction are on the scale of the respective parameter; donor-referenced estimates are in standard deviations of the sex-specific donor reference and are not numerically comparable with unstandardized estimates. The continuous follow-up model (*) estimates a per-year coefficient and is not plotted in Figure 3C. For ex vivo analyses, curve-level sex and group effects are mixed-model coefficients, constant across bradykinin concentrations; estimates at the maximal concentration (10⁻⁶ M) are mean differences with Welch t-test P values; NO component comparisons use Mann–Whitney U tests, and the group × sex estimate for the NO component is from ordinary least squares. The passive stiffness estimate is the difference in medians. NO, nitric oxide; pp, percentage points.

**Supplementary Table S3. Baseline analytes versus change in lnRHI.**

| **Analyte** | **n** | **ρ** | **P** | **P (BH)** |
| --- | --- | --- | --- | --- |
| Total cholesterol | 70 | +0.24 | 0.050 | 0.321 |
| Calcium | 70 | +0.22 | 0.070 | 0.321 |
| HDL cholesterol | 70 | +0.21 | 0.076 | 0.321 |
| Apolipoprotein B | 69 | +0.21 | 0.080 | 0.321 |
| Homocysteine | 70 | −0.17 | 0.170 | 0.445 |
| Lipoprotein(a) | 66 | +0.17 | 0.180 | 0.445 |
| Phosphate | 70 | +0.16 | 0.195 | 0.445 |
| Albumin | 70 | −0.14 | 0.231 | 0.462 |
| Creatinine | 70 | −0.09 | 0.452 | 0.737 |
| 25-OH vitamin D | 70 | +0.09 | 0.461 | 0.737 |
| hs-Troponin T | 69 | −0.06 | 0.640 | 0.862 |
| Interleukin-6 | 46 | +0.06 | 0.677 | 0.862 |
| hs-CRP | 70 | +0.04 | 0.718 | 0.862 |
| Triglycerides | 70 | +0.04 | 0.763 | 0.862 |
| Parathyroid hormone | 68 | +0.03 | 0.808 | 0.862 |
| HbA1c | 68 | +0.00 | 0.985 | 0.985 |

Spearman correlations across the 16 baseline analytes listed in Table 1. Values below the limit of quantification were assigned half that limit (hs-CRP, Lp(a), interleukin-6). P (BH), Benjamini–Hochberg-corrected across the 16 analytes. No analyte was associated with the change in lnRHI after correction.

**Supplementary Table S4. Uremic toxin and tryptophan-metabolite panel versus change in lnRHI.**

| **Analyte** | **n** | **ρ** | **P** | **P (BH)** |
| --- | --- | --- | --- | --- |
| p-Cresyl sulphate | 29 | +0.29 | 0.125 | 0.874 |
| Hippuric acid | 29 | −0.24 | 0.204 | 0.874 |
| Kynurenic acid | 29 | −0.23 | 0.235 | 0.874 |
| Tyrosine | 29 | +0.21 | 0.265 | 0.874 |
| Indole-3-acetic acid | 29 | −0.20 | 0.293 | 0.874 |
| Phenylalanine | 29 | +0.17 | 0.368 | 0.874 |
| Indoxyl sulphate | 29 | −0.13 | 0.506 | 0.874 |
| Phenyl sulphate | 29 | −0.12 | 0.526 | 0.874 |
| p-Cresyl glucuronide | 29 | +0.12 | 0.550 | 0.874 |
| TMAO | 29 | +0.11 | 0.583 | 0.874 |
| CMPF | 29 | +0.08 | 0.670 | 0.891 |
| Phenylacetylglutamine | 29 | +0.07 | 0.738 | 0.891 |
| Phenyl glucuronide | 29 | +0.06 | 0.772 | 0.891 |
| Kynurenine | 29 | −0.02 | 0.915 | 0.951 |
| Tryptophan | 29 | +0.01 | 0.951 | 0.951 |

Spearman correlations in the 29 recipients with a measured panel (22 men, 7 women; donor→recipient F→M 13, M→M 8, M→F 4, F→F 3). The subset is predominantly male and cannot support inference by sex. P (BH), Benjamini–Hochberg-corrected across the 15 analytes. Baseline indoxyl sulphate was not associated with the change in lnRHI (ρ = −0.13, P = 0.51); its association with the follow-up RHI value (ρ = −0.43, P = 0.019) concerns a different endpoint and is reported separately in the main text. Indoxyl sulphate concentrations did not differ between the sexes (P = 1.00).

**Supplementary Table S5. Ex vivo relaxation to bradykinin by sex in living donors and recipients.**

|  | **Living donors** | | **Recipients** | |
| --- | --- | --- | --- | --- |
|  | **Men (n = 20)** | **Women (n = 26)** | **Men (n = 23)** | **Women (n = 14)** |
| *Relaxation without L-NAME, %* | | | | |
| Bradykinin 10⁻¹¹ M | 7.9 ± 8.3 | 5.5 ± 4.9 | 5.3 ± 5.4 | 4.5 ± 4.1 |
| Bradykinin 10⁻¹⁰ M | 14.3 ± 9.4 | 16.0 ± 14.1 | 15.0 ± 12.7 | 11.4 ± 9.5 |
| Bradykinin 10⁻⁹ M | 22.1 ± 13.5 | 26.0 ± 19.9 | 22.3 ± 14.7 | 20.0 ± 14.3 |
| Bradykinin 10⁻⁸ M | 45.3 ± 25.3 | 45.6 ± 20.4 | 41.2 ± 18.9 | 40.3 ± 26.1 |
| Bradykinin 10⁻⁷ M | 71.0 ± 19.7 | 70.5 ± 16.6 | 63.2 ± 20.7 | 63.2 ± 25.1 |
| Bradykinin 10⁻⁶ M | 78.7 ± 14.8 | 77.2 ± 12.6 | 75.0 ± 17.2 | 77.1 ± 22.4 |
| *Relaxation with L-NAME, %* | | | | |
| n | 20 | 25 | 21 | 14 |
| Bradykinin 10⁻¹¹ M | 3.6 ± 4.6 | 4.6 ± 6.1 | 4.9 ± 4.4 | 4.0 ± 4.0 |
| Bradykinin 10⁻¹⁰ M | 14.2 ± 16.9 | 10.0 ± 9.9 | 13.9 ± 12.9 | 17.4 ± 14.6 |
| Bradykinin 10⁻⁹ M | 20.4 ± 22.5 | 18.6 ± 16.1 | 22.7 ± 17.7 | 29.5 ± 21.9 |
| Bradykinin 10⁻⁸ M | 27.7 ± 24.7 | 30.3 ± 19.6 | 33.6 ± 22.4 | 44.6 ± 24.9 |
| Bradykinin 10⁻⁷ M | 51.2 ± 23.8 | 51.6 ± 20.1 | 47.7 ± 24.9 | 55.1 ± 29.3 |
| Bradykinin 10⁻⁶ M | 61.6 ± 21.8 | 62.1 ± 18.3 | 62.1 ± 24.6 | 71.3 ± 26.2 |
| *Components at 10⁻⁶ M, % of total relaxation* | | | | |
| NO | 24.5 ± 19.2 | 20.5 ± 17.1 | 21.2 ± 24.9 | 11.2 ± 14.0 |
| EDH | 75.5 ± 19.2 | 79.5 ± 17.1 | 78.8 ± 24.9 | 88.8 ± 14.0 |
| *Men versus women* | | | | |
| Sex effect, pp (95% CI) | −0.3 (−7.3 to +6.8) | | +0.9 (−7.7 to +9.5) | |
| P, sex effect | 0.944 | | 0.834 | |
| P, concentration × sex | 0.765 (0.159) | | 0.935 (0.594) | |
| Difference at 10⁻⁶ M, pp (95% CI) | +1.5 (−6.8 to +9.9) | | −2.1 (−16.5 to +12.4) | |
| P, difference at 10⁻⁶ M | 0.710 | | 0.770 | |
| Smallest detectable difference, pp | 11.3 | | 18.3 | |
| P, sex effect with L-NAME | 0.953 | | 0.276 | |
| P, concentration × sex with L-NAME | 0.810 (0.297) | | 0.389 (0.042) | |
| P, NO component | 0.560 | | 0.152 | |

Values are mean ± SD of relaxation to cumulative bradykinin, expressed as percentage reversal of norepinephrine-induced tone. Recipient vessels were obtained at transplantation and donor vessels at nephrectomy. Sex effects are men − women, from linear mixed-effects models with bradykinin concentration as a categorical fixed effect and a random vessel intercept, fitted by maximum likelihood; concentration × sex P values are likelihood-ratio tests of the five interaction terms, with the smallest individual term in parentheses. Differences at 10⁻⁶ M are mean differences with Welch t-test 95% CI and P; the smallest detectable difference at 10⁻⁶ M corresponds to 80% power at α = 0.05. The NO component is the L-NAME-inhibitable fraction of relaxation at 10⁻⁶ M, set to zero where relaxation with L-NAME equaled or exceeded relaxation without it (six donor and five recipient vessels), and the EDH component is its complement; P by Mann–Whitney U test. Vessels without a complete L-NAME curve were excluded from the L-NAME rows (one donor, two recipients). Female donors were older than male donors (52.5 ± 10.8 vs 45.3 ± 10.1 years; P = 0.024), and adjustment for age left the donor sex effect unchanged (P = 0.948). Group × sex interaction: relaxation P = 0.835; NO component P = 0.510. EDH, endothelium-dependent hyperpolarization; NO, nitric oxide; pp, percentage points.
